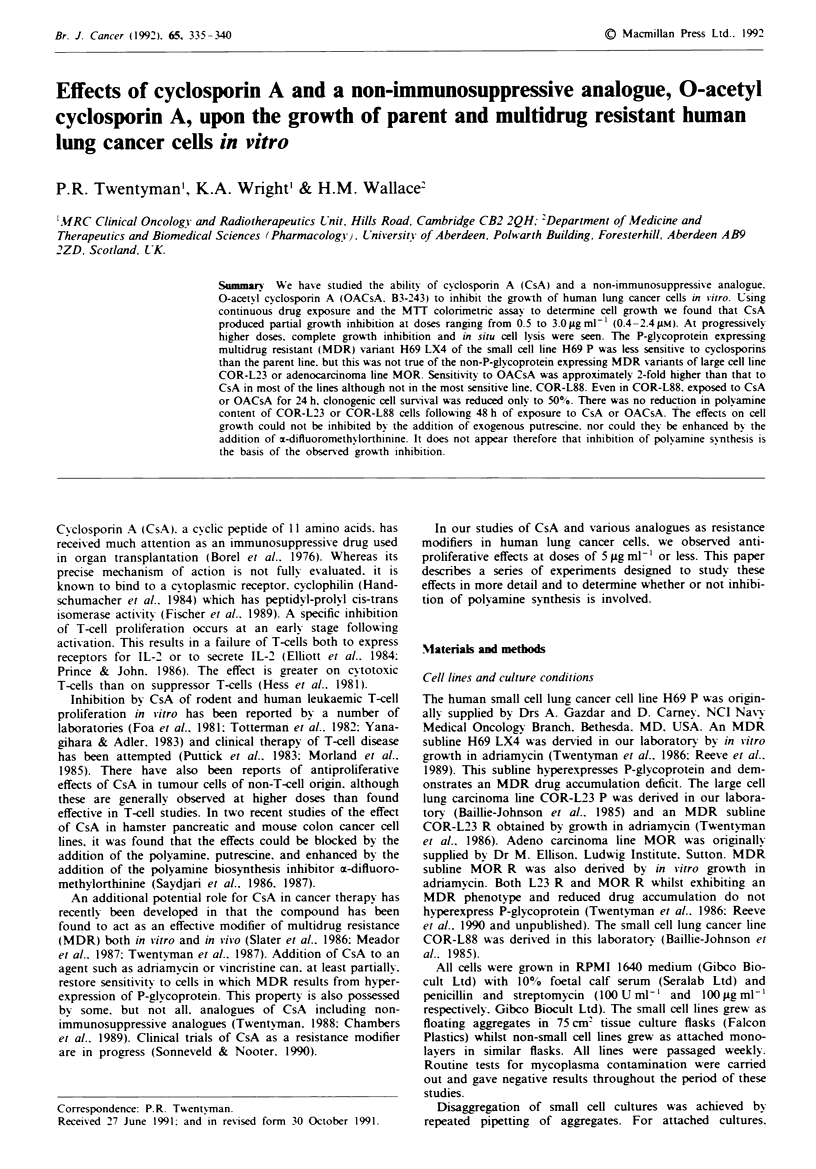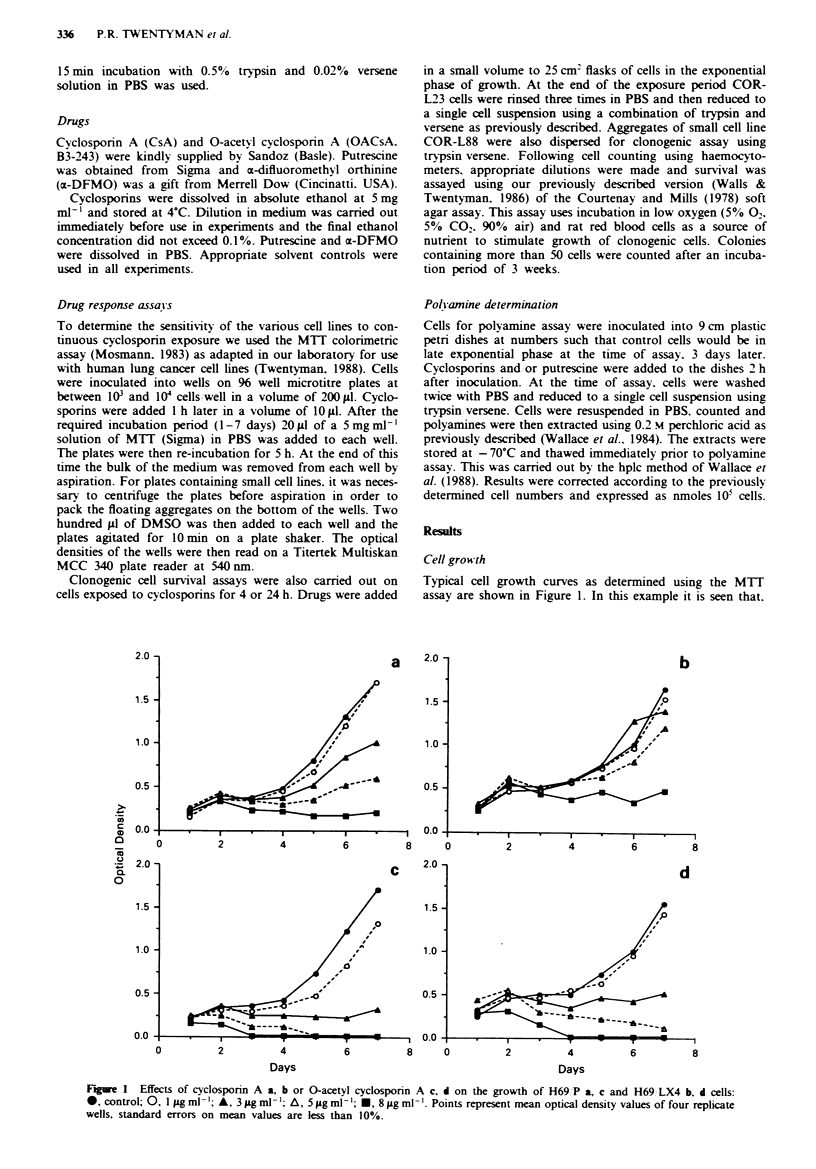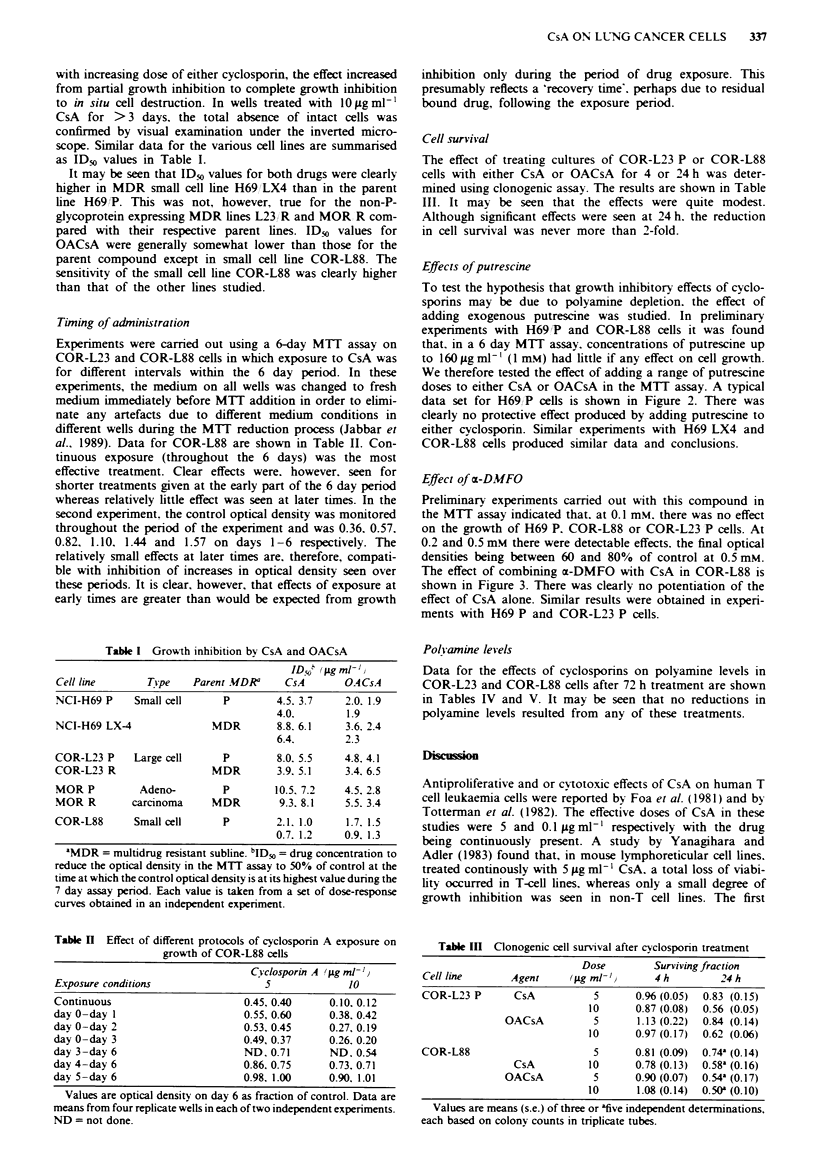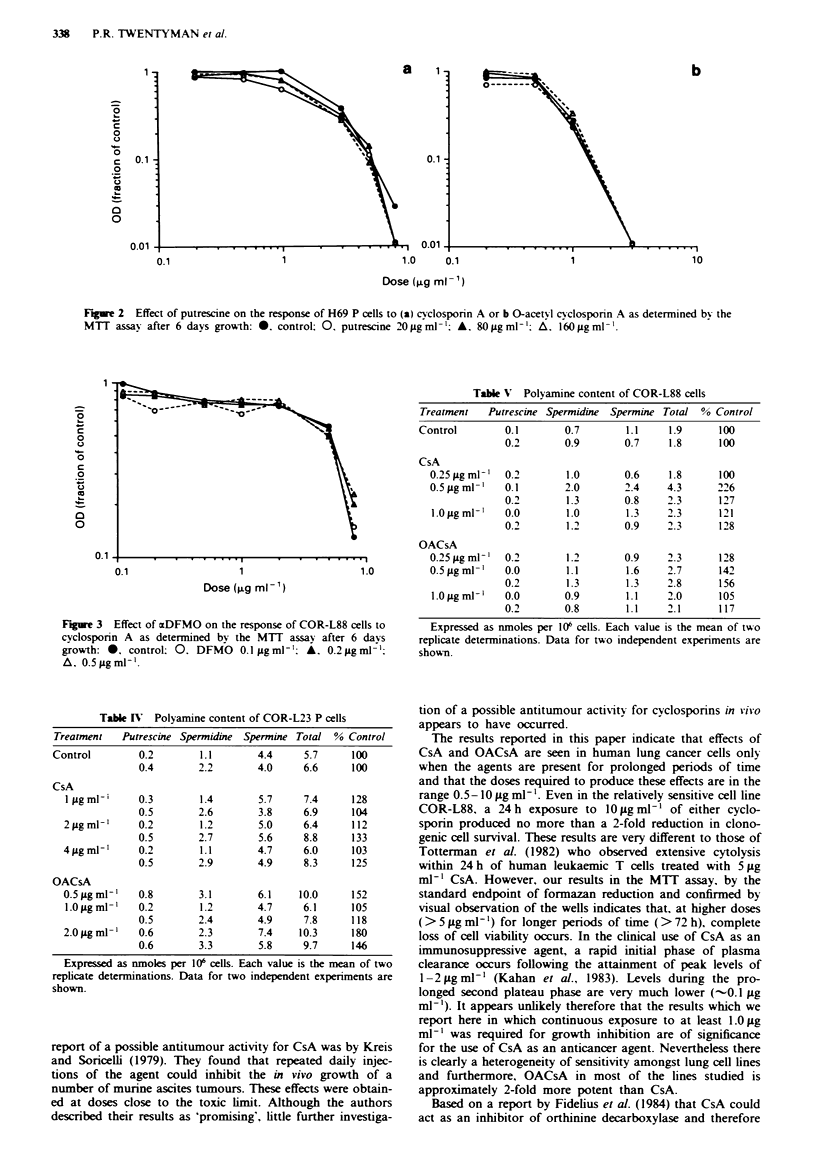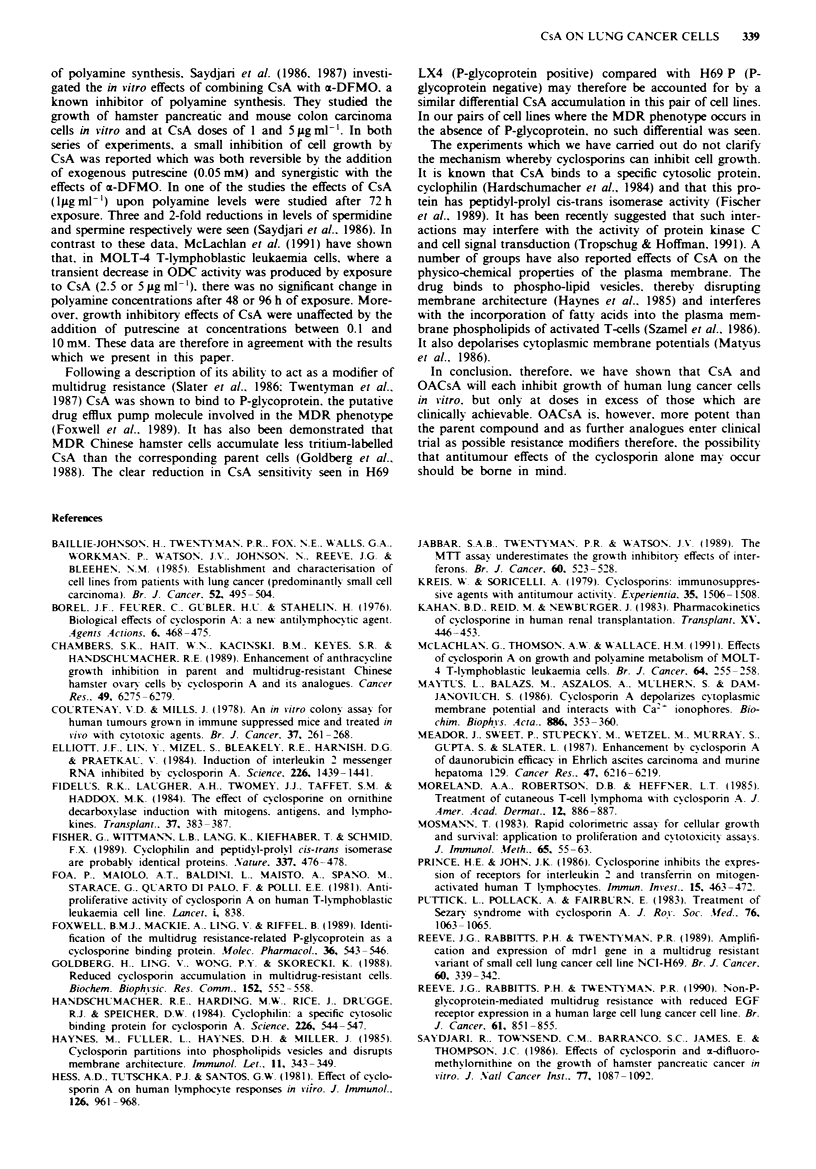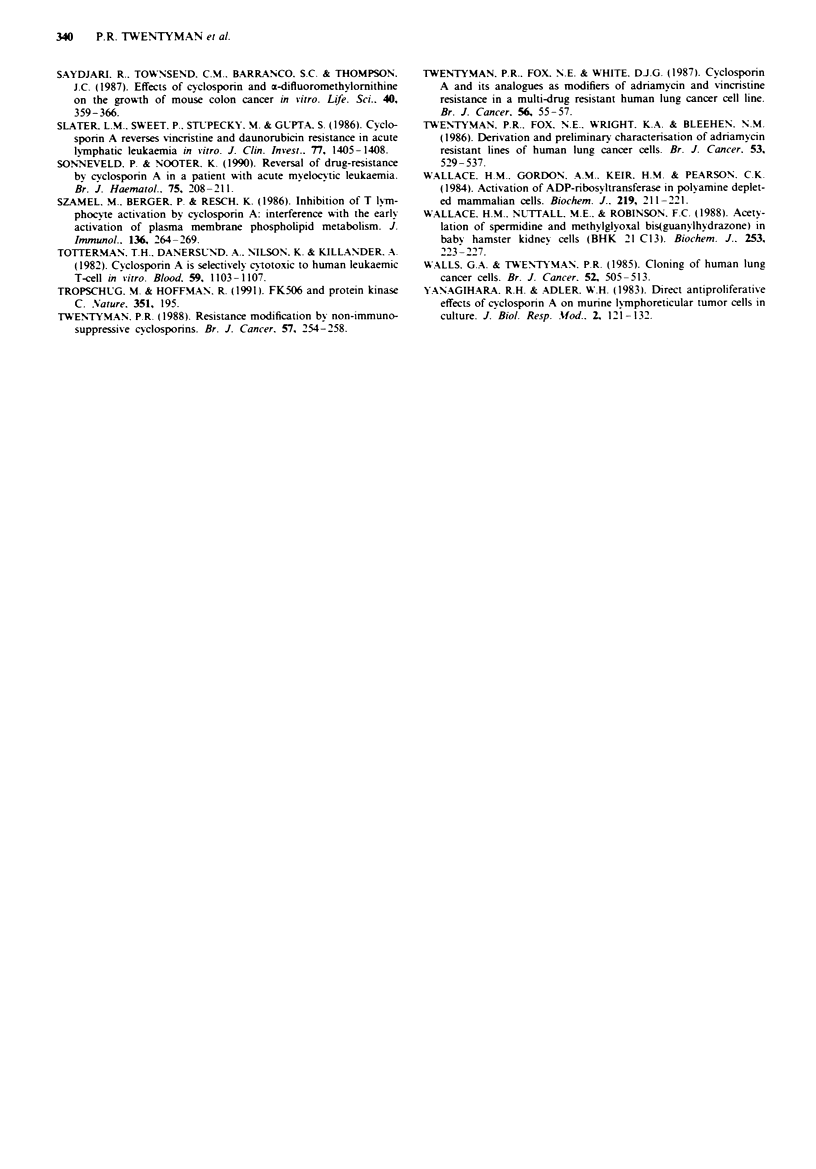# Effects of cyclosporin A and a non-immunosuppressive analogue, O-acetyl cyclosporin A, upon the growth of parent and multidrug resistant human lung cancer cells in vitro.

**DOI:** 10.1038/bjc.1992.68

**Published:** 1992-03

**Authors:** P. R. Twentyman, K. A. Wright, H. M. Wallace

**Affiliations:** MRC Clinical Oncology and Radiotherapeutics Unit, Cambridge, UK.

## Abstract

We have studied the ability of cyclosporin A (CsA) and a non-immunosuppressive analogue, O-acetyl cyclosporin A (OACsA, B3-243) to inhibit the growth of human lung cancer cells in vitro. Using continuous drug exposure and the MTT colorimetric assay to determine cell growth we found that CsA produced partial growth inhibition at doses ranging from 0.5 to 3.0 micrograms ml-1 (0.4-2.4 microM). At progressively higher doses, complete growth inhibition and in situ cell lysis were seen. The P-glycoprotein expressing multidrug resistant (MDR) variant H69/LX4 of the small cell line H69/P was less sensitive to cyclosporins than the parent line, but this was not true of the non-P-glycoprotein expressing MDR variants of large cell line COR-L23 or adenocarcinoma line MOR. Sensitivity to OACsA was approximately 2-fold higher than that to CsA in most of the lines although not in the most sensitive line, COR-L88. Even in COR-L88, exposed to CsA or OACsA for 24 h, clonogenic cell survival was reduced only to 50%. There was no reduction in polyamine content of COR-L23 or COR-L88 cells following 48 h of exposure to CsA or OACsA. The effects on cell growth could not be inhibited by the addition of exogenous putrescine, nor could they be enhanced by the addition of alpha-difluoromethylorthinine. It does not appear therefore that inhibition of polyamine synthesis is the basis of the observed growth inhibition.


Br. J. Cancer (1992), 65, 335-340 © Macmillan Press Ltd., 1992

### Effects of cyclosporin A and a non-immunosuppressive analogue, O-acetyl cyclosporin A, upon the growth of parent and multidrug resistant human lung cancer cells *in vitro*

P.R. Twentyman[1], K.A. Wright[1] & H.M. Wallace[2]

[1]*MRC Clinical Oncology and Radiotherapeutics Unit, Hills Road, Cambridge CB2 2QH;* [2]*Department of Medicine and Therapeutics and Biomedical Sciences (Pharmacology), University of Aberdeen, Polwarth Building, Foresterhill, Aberdeen AB9 2ZD, Scotland, UK.*

**Summary** We have studied the ability of cyclosporin A (CsA) and a non-immunosuppressive analogue, O-acetyl cyclosporin A (OACsA, B3-243) to inhibit the growth of human lung cancer cells *in vitro*. Using continuous drug exposure and the MTT colorimetric assay to determine cell growth we found that CsA produced partial growth inhibition at doses ranging from 0.5 to 3.0 $\mu g\ ml^{-1}$ (0.4–2.4 $\mu M$). At progressively higher doses, complete growth inhibition and *in situ* cell lysis were seen. The P-glycoprotein expressing multidrug resistant (MDR) variant H69 LX4 of the small cell line H69 P was less sensitive to cyclosporins than the parent line, but this was not true of the non-P-glycoprotein expressing MDR variants of large cell line COR-L23 or adenocarcinoma line MOR. Sensitivity to OACsA was approximately 2-fold higher than that to CsA in most of the lines although not in the most sensitive line, COR-L88. Even in COR-L88, exposed to CsA or OACsA for 24 h, clonogenic cell survival was reduced only to 50%. There was no reduction in polyamine content of COR-L23 or COR-L88 cells following 48 h of exposure to CsA or OACsA. The effects on cell growth could not be inhibited by the addition of exogenous putrescine, nor could they be enhanced by the addition of α-difluoromethylorthinine. It does not appear therefore that inhibition of polyamine synthesis is the basis of the observed growth inhibition.

Cyclosporin A (CsA), a cyclic peptide of 11 amino acids, has received much attention as an immunosuppressive drug used in organ transplantation (Borel *et al.*, 1976). Whereas its precise mechanism of action is not fully evaluated, it is known to bind to a cytoplasmic receptor, cyclophilin (Handschumacher *et al.*, 1984) which has peptidyl-prolyl cis-trans isomerase activity (Fischer *et al.*, 1989). A specific inhibition of T-cell proliferation occurs at an early stage following activation. This results in a failure of T-cells both to express receptors for IL-2 or to secrete IL-2 (Elliott *et al.*, 1984; Prince & John, 1986). The effect is greater on cytotoxic T-cells than on suppressor T-cells (Hess *et al.*, 1981).

Inhibition by CsA of rodent and human leukaemic T-cell proliferation *in vitro* has been reported by a number of laboratories (Foa *et al.*, 1981; Totterman *et al.*, 1982; Yanagihara & Adler, 1983) and clinical therapy of T-cell disease has been attempted (Puttick *et al.*, 1983; Morland *et al.*, 1985). There have also been reports of antiproliferative effects of CsA in tumour cells of non-T-cell origin, although these are generally observed at higher doses than found effective in T-cell studies. In two recent studies of the effect of CsA in hamster pancreatic and mouse colon cancer cell lines, it was found that the effects could be blocked by the addition of the polyamine, putrescine, and enhanced by the addition of the polyamine biosynthesis inhibitor α-difluoromethylorthinine (Saydjari *et al.*, 1986, 1987).

An additional potential role for CsA in cancer therapy has recently been developed in that the compound has been found to act as an effective modifier of multidrug resistance (MDR) both *in vitro* and *in vivo* (Slater *et al.*, 1986; Meador *et al.*, 1987; Twentyman *et al.*, 1987). Addition of CsA to an agent such as adriamycin or vincristine can, at least partially, restore sensitivity to cells in which MDR results from hyperexpression of P-glycoprotein. This property is also possessed by some, but not all, analogues of CsA including non-immunosuppressive analogues (Twentyman, 1988; Chambers *et al.*, 1989). Clinical trials of CsA as a resistance modifier are in progress (Sonneveld & Nooter, 1990).

Correspondence: P.R. Twentyman.
Received 27 June 1991; and in revised form 30 October 1991.

In our studies of CsA and various analogues as resistance modifiers in human lung cancer cells, we observed anti-proliferative effects at doses of 5 $\mu g\ ml^{-1}$ or less. This paper describes a series of experiments designed to study these effects in more detail and to determine whether or not inhibition of polyamine synthesis is involved.

### Materials and methods

### *Cell lines and culture conditions*

The human small cell lung cancer cell line H69 P was originally supplied by Drs A. Gazdar and D. Carney, NCI Navy Medical Oncology Branch, Bethesda, MD, USA. An MDR subline H69 LX4 was dervied in our laboratory by *in vitro* growth in adriamycin (Twentyman *et al.*, 1986; Reeve *et al.*, 1989). This subline hyperexpresses P-glycoprotein and demonstrates an MDR drug accumulation deficit. The large cell lung carcinoma line COR-L23 P was derived in our laboratory (Baillie-Johnson *et al.*, 1985) and an MDR subline COR-L23 R obtained by growth in adriamycin (Twentyman *et al.*, 1986). Adeno carcinoma line MOR was originally supplied by Dr M. Ellison, Ludwig Institute, Sutton. MDR subline MOR R was also derived by *in vitro* growth in adriamycin. Both L23 R and MOR R whilst exhibiting an MDR phenotype and reduced drug accumulation do not hyperexpress P-glycoprotein (Twentyman *et al.*, 1986; Reeve *et al.*, 1990 and unpublished). The small cell lung cancer line COR-L88 was derived in this laboratory (Baillie-Johnson *et al.*, 1985).

All cells were grown in RPMI 1640 medium (Gibco Biocult Ltd) with 10% foetal calf serum (Seralab Ltd) and penicillin and streptomycin (100 $U\ ml^{-1}$ and 100 $\mu g\ ml^{-1}$ respectively, Gibco Biocult Ltd). The small cell lines grew as floating aggregates in 75 $cm^2$ tissue culture flasks (Falcon Plastics) whilst non-small cell lines grew as attached monolayers in similar flasks. All lines were passaged weekly. Routine tests for mycoplasma contamination were carried out and gave negative results throughout the period of these studies.

Disaggregation of small cell cultures was achieved by repeated pipetting of aggregates. For attached cultures,

15 min incubation with 0.5% trypsin and 0.02% versene solution in PBS was used.

*Drugs*

Cyclosporin A (CsA) and O-acetyl cyclosporin A (OACsA, B3-243) were kindly supplied by Sandoz (Basle). Putrescine was obtained from Sigma and α-difluoromethyl orthinine (α-DFMO) was a gift from Merrell Dow (Cincinatti, USA).

Cyclosporins were dissolved in absolute ethanol at 5 mg $ml^{-1}$ and stored at 4°C. Dilution in medium was carried out immediately before use in experiments and the final ethanol concentration did not exceed 0.1%. Putrescine and α-DFMO were dissolved in PBS. Appropriate solvent controls were used in all experiments.

*Drug response assays*

To determine the sensitivity of the various cell lines to continuous cyclosporin exposure we used the MTT colorimetric assay (Mosmann, 1983) as adapted in our laboratory for use with human lung cancer cell lines (Twentyman, 1988). Cells were inoculated into wells on 96 well microtitre plates at between $10^3$ and $10^4$ cells/well in a volume of 200 µl. Cyclosporins were added 1 h later in a volume of 10 µl. After the required incubation period (1–7 days) 20 µl of a 5 mg $ml^{-1}$ solution of MTT (Sigma) in PBS was added to each well. The plates were then re-incubation for 5 h. At the end of this time the bulk of the medium was removed from each well by aspiration. For plates containing small cell lines, it was necessary to centrifuge the plates before aspiration in order to pack the floating aggregates on the bottom of the wells. Two hundred µl of DMSO was then added to each well and the plates agitated for 10 min on a plate shaker. The optical densities of the wells were then read on a Titertek Multiskan MCC 340 plate reader at 540 nm.

Clonogenic cell survival assays were also carried out on cells exposed to cyclosporins for 4 or 24 h. Drugs were added in a small volume to 25 $cm^2$ flasks of cells in the exponential phase of growth. At the end of the exposure period COR-L23 cells were rinsed three times in PBS and then reduced to a single cell suspension using a combination of trypsin and versene as previously described. Aggregates of small cell line COR-L88 were also dispersed for clonogenic assay using trypsin/versene. Following cell counting using haemocytometers, appropriate dilutions were made and survival was assayed using our previously described version (Walls & Twentyman, 1986) of the Courtenay and Mills (1978) soft agar assay. This assay uses incubation in low oxygen (5% $O_2$, 5% $CO_2$, 90% air) and rat red blood cells as a source of nutrient to stimulate growth of clonogenic cells. Colonies containing more than 50 cells were counted after an incubation period of 3 weeks.

*Polyamine determination*

Cells for polyamine assay were inoculated into 9 cm plastic petri dishes at numbers such that control cells would be in late exponential phase at the time of assay, 3 days later. Cyclosporins and/or putrescine were added to the dishes 2 h after inoculation. At the time of assay, cells were washed twice with PBS and reduced to a single cell suspension using trypsin/versene. Cells were resuspended in PBS, counted and polyamines were then extracted using 0.2 M perchloric acid as previously described (Wallace *et al.*, 1984). The extracts were stored at −70°C and thawed immediately prior to polyamine assay. This was carried out by the hplc method of Wallace *et al.* (1988). Results were corrected according to the previously determined cell numbers and expressed as nmoles/$10^5$ cells.

### Results

*Cell growth*

Typical cell growth curves as determined using the MTT assay are shown in Figure 1. In this example it is seen that,

**Figure 1** Effects of cyclosporin A **a**, **b** or O-acetyl cyclosporin A **c**, **d** on the growth of H69/P **a**, **c** and H69/LX4 **b**, **d** cells: ●, control; ○, 1 µg $ml^{-1}$; ▲, 3 µg $ml^{-1}$; △, 5 µg $ml^{-1}$; ■, 8 µg $ml^{-1}$. Points represent mean optical density values of four replicate wells, standard errors on mean values are less than 10%.

with increasing dose of either cyclosporin, the effect increased from partial growth inhibition to complete growth inhibition to *in situ* cell destruction. In wells treated with 10 μg ml$^{-1}$ CsA for >3 days, the total absence of intact cells was confirmed by visual examination under the inverted microscope. Similar data for the various cell lines are summarised as $ID_{50}$ values in Table I.

It may be seen that $ID_{50}$ values for both drugs were clearly higher in MDR small cell line H69/LX4 than in the parent line H69/P. This was not, however, true for the non-P-glycoprotein expressing MDR lines L23/R and MOR/R compared with their respective parent lines. $ID_{50}$ values for OACsA were generally somewhat lower than those for the parent compound except in small cell line COR-L88. The sensitivity of the small cell line COR-L88 was clearly higher than that of the other lines studied.

*Timing of administration*

Experiments were carried out using a 6-day MTT assay on COR-L23 and COR-L88 cells in which exposure to CsA was for different intervals within the 6 day period. In these experiments, the medium on all wells was changed to fresh medium immediately before MTT addition in order to eliminate any artefacts due to different medium conditions in different wells during the MTT reduction process (Jabbar *et al.*, 1989). Data for COR-L88 are shown in Table II. Continuous exposure (throughout the 6 days) was the most effective treatment. Clear effects were, however, seen for shorter treatments given at the early part of the 6 day period whereas relatively little effect was seen at later times. In the second experiment, the control optical density was monitored throughout the period of the experiment and was 0.36, 0.57, 0.82, 1.10, 1.44 and 1.57 on days 1–6 respectively. The relatively small effects at later times are, therefore, compatible with inhibition of increases in optical density seen over these periods. It is clear, however, that effects of exposure at early times are greater than would be expected from growth inhibition only during the period of drug exposure. This presumably reflects a 'recovery time', perhaps due to residual bound drug, following the exposure period.

*Cell survival*

The effect of treating cultures of COR-L23 P or COR-L88 cells with either CsA or OACsA for 4 or 24 h was determined using clonogenic assay. The results are shown in Table III. It may be seen that the effects were quite modest. Although significant effects were seen at 24 h, the reduction in cell survival was never more than 2-fold.

*Effects of putrescine*

To test the hypothesis that growth inhibitory effects of cyclosporins may be due to polyamine depletion, the effect of adding exogenous putrescine was studied. In preliminary experiments with H69/P and COR-L88 cells it was found that, in a 6 day MTT assay, concentrations of putrescine up to 160 μg ml$^{-1}$ (1 mM) had little if any effect on cell growth. We therefore tested the effect of adding a range of putrescine doses to either CsA or OACsA in the MTT assay. A typical data set for H69/P cells is shown in Figure 2. There was clearly no protective effect produced by adding putrescine to either cyclosporin. Similar experiments with H69/LX4 and COR-L88 cells produced similar data and conclusions.

*Effect of α-DMFO*

Preliminary experiments carried out with this compound in the MTT assay indicated that, at 0.1 mM, there was no effect on the growth of H69/P, COR-L88 or COR-L23/P cells. At 0.2 and 0.5 mM there were detectable effects, the final optical densities being between 60 and 80% of control at 0.5 mM. The effect of combining α-DMFO with CsA in COR-L88 is shown in Figure 3. There was clearly no potentiation of the effect of CsA alone. Similar results were obtained in experiments with H69/P and COR-L23/P cells.

*Polyamine levels*

Data for the effects of cyclosporins on polyamine levels in COR-L23 and COR-L88 cells after 72 h treatment are shown in Tables IV and V. It may be seen that no reductions in polyamine levels resulted from any of these treatments.

### Discussion

Antiproliferative and/or cytotoxic effects of CsA on human T cell leukaemia cells were reported by Foa *et al.* (1981) and by Totterman *et al.* (1982). The effective doses of CsA in these studies were 5 and 0.1 μg ml$^{-1}$ respectively with the drug being continuously present. A study by Yanagihara and Adler (1983) found that, in mouse lymphoreticular cell lines, treated continously with 5 μg ml$^{-1}$ CsA, a total loss of viability occurred in T-cell lines, whereas only a small degree of growth inhibition was seen in non-T cell lines. The first

**Table I** Growth inhibition by CsA and OACsA

| Cell line | Type | Parent/MDR[a] | $ID_{50}$[b] (μg ml$^{-1}$) CsA | OACsA |
|---|---|---|---|---|
| NCI-H69 P | Small cell | P | 4.5, 3.7<br>4.0, | 2.0, 1.9<br>1.9 |
| NCI-H69 LX-4 | | MDR | 8.8, 6.1<br>6.4, | 3.6, 2.4<br>2.3 |
| COR-L23 P | Large cell | P | 8.0, 5.5 | 4.8, 4.1 |
| COR-L23 R | | MDR | 3.9, 5.1 | 3.4, 6.5 |
| MOR P | Adeno- | P | 10.5, 7.2 | 4.5, 2.8 |
| MOR R | carcinoma | MDR | 9.3, 8.1 | 5.5, 3.4 |
| COR-L88 | Small cell | P | 2.1, 1.0<br>0.7, 1.2 | 1.7, 1.5<br>0.9, 1.3 |

[a]MDR = multidrug resistant subline. [b]$ID_{50}$ = drug concentration to reduce the optical density in the MTT assay to 50% of control at the time at which the control optical density is at its highest value during the 7 day assay period. Each value is taken from a set of dose-response curves obtained in an independent experiment.

**Table II** Effect of different protocols of cyclosporin A exposure on growth of COR-L88 cells

| Exposure conditions | Cyclosporin A (μg ml$^{-1}$) 5 | 10 |
|---|---|---|
| Continuous | 0.45, 0.40 | 0.10, 0.12 |
| day 0–day 1 | 0.55, 0.60 | 0.38, 0.42 |
| day 0–day 2 | 0.53, 0.45 | 0.27, 0.19 |
| day 0–day 3 | 0.49, 0.37 | 0.26, 0.20 |
| day 3–day 6 | ND, 0.71 | ND, 0.54 |
| day 4–day 6 | 0.86, 0.75 | 0.73, 0.71 |
| day 5–day 6 | 0.98, 1.00 | 0.90, 1.01 |

Values are optical density on day 6 as fraction of control. Data are means from four replicate wells in each of two independent experiments. ND = not done.

**Table III** Clonogenic cell survival after cyclosporin treatment

| Cell line | Agent | Dose (μg ml$^{-1}$) | Surviving fraction 4 h | 24 h |
|---|---|---|---|---|
| COR-L23 P | CsA | 5 | 0.96 (0.05) | 0.83 (0.15) |
| | | 10 | 0.87 (0.08) | 0.56 (0.05) |
| | OACsA | 5 | 1.13 (0.22) | 0.84 (0.14) |
| | | 10 | 0.97 (0.17) | 0.62 (0.06) |
| COR-L88 | | 5 | 0.81 (0.09) | 0.74[a] (0.14) |
| | CsA | 10 | 0.78 (0.13) | 0.58[a] (0.16) |
| | OACsA | 5 | 0.90 (0.07) | 0.54[a] (0.17) |
| | | 10 | 1.08 (0.14) | 0.50[a] (0.10) |

Values are means (s.e.) of three or [a]five independent determinations, each based on colony counts in triplicate tubes.

**Figure 2** Effect of putrescine on the response of H69 P cells to (a) cyclosporin A or b O-acetyl cyclosporin A as determined by the MTT assay after 6 days growth: ●, control; O, putrescine 20 μg ml$^{-1}$; ▲, 80 μg ml$^{-1}$; Δ, 160 μg ml$^{-1}$.

**Figure 3** Effect of αDFMO on the response of COR-L88 cells to cyclosporin A as determined by the MTT assay after 6 days growth: ●, control; O, DFMO 0.1 μg ml$^{-1}$; ▲, 0.2 μg ml$^{-1}$; Δ, 0.5 μg ml$^{-1}$.

**Table V** Polyamine content of COR-L88 cells

| *Treatment* | *Putrescine* | *Spermidine* | *Spermine* | *Total* | *% Control* |
|---|---|---|---|---|---|
| Control | 0.1 | 0.7 | 1.1 | 1.9 | 100 |
| | 0.2 | 0.9 | 0.7 | 1.8 | 100 |
| CsA | | | | | |
| 0.25 μg ml$^{-1}$ | 0.2 | 1.0 | 0.6 | 1.8 | 100 |
| 0.5 μg ml$^{-1}$ | 0.1 | 2.0 | 2.4 | 4.3 | 226 |
| | 0.2 | 1.3 | 0.8 | 2.3 | 127 |
| 1.0 μg ml$^{-1}$ | 0.0 | 1.0 | 1.3 | 2.3 | 121 |
| | 0.2 | 1.2 | 0.9 | 2.3 | 128 |
| OACsA | | | | | |
| 0.25 μg ml$^{-1}$ | 0.2 | 1.2 | 0.9 | 2.3 | 128 |
| 0.5 μg ml$^{-1}$ | 0.0 | 1.1 | 1.6 | 2.7 | 142 |
| | 0.2 | 1.3 | 1.3 | 2.8 | 156 |
| 1.0 μg ml$^{-1}$ | 0.0 | 0.9 | 1.1 | 2.0 | 105 |
| | 0.2 | 0.8 | 1.1 | 2.1 | 117 |

Expressed as nmoles per $10^6$ cells. Each value is the mean of two replicate determinations. Data for two independent experiments are shown.

**Table IV** Polyamine content of COR-L23 P cells

| *Treatment* | *Putrescine* | *Spermidine* | *Spermine* | *Total* | *% Control* |
|---|---|---|---|---|---|
| Control | 0.2 | 1.1 | 4.4 | 5.7 | 100 |
| | 0.4 | 2.2 | 4.0 | 6.6 | 100 |
| CsA | | | | | |
| 1 μg ml$^{-1}$ | 0.3 | 1.4 | 5.7 | 7.4 | 128 |
| | 0.5 | 2.6 | 3.8 | 6.9 | 104 |
| 2 μg ml$^{-1}$ | 0.2 | 1.2 | 5.0 | 6.4 | 112 |
| | 0.5 | 2.7 | 5.6 | 8.8 | 133 |
| 4 μg ml$^{-1}$ | 0.2 | 1.1 | 4.7 | 6.0 | 103 |
| | 0.5 | 2.9 | 4.9 | 8.3 | 125 |
| OACsA | | | | | |
| 0.5 μg ml$^{-1}$ | 0.8 | 3.1 | 6.1 | 10.0 | 152 |
| 1.0 μg ml$^{-1}$ | 0.2 | 1.2 | 4.7 | 6.1 | 105 |
| | 0.5 | 2.4 | 4.9 | 7.8 | 118 |
| 2.0 μg ml$^{-1}$ | 0.6 | 2.3 | 7.4 | 10.3 | 180 |
| | 0.6 | 3.3 | 5.8 | 9.7 | 146 |

Expressed as nmoles per $10^6$ cells. Each value is the mean of two replicate determinations. Data for two independent experiments are shown.

report of a possible antitumour activity for CsA was by Kreis and Soricelli (1979). They found that repeated daily injections of the agent could inhibit the *in vivo* growth of a number of murine ascites tumours. These effects were obtained at doses close to the toxic limit. Although the authors described their results as 'promising', little further investigation of a possible antitumour activity for cyclosporins *in vivo* appears to have occurred.

The results reported in this paper indicate that effects of CsA and OACsA are seen in human lung cancer cells only when the agents are present for prolonged periods of time and that the doses required to produce these effects are in the range 0.5–10 μg ml$^{-1}$. Even in the relatively sensitive cell line COR-L88, a 24 h exposure to 10 μg ml$^{-1}$ of either cyclosporin produced no more than a 2-fold reduction in clonogenic cell survival. These results are very different to those of Totterman *et al.* (1982) who observed extensive cytolysis within 24 h of human leukaemic T cells treated with 5 μg ml$^{-1}$ CsA. However, our results in the MTT assay, by the standard endpoint of formazan reduction and confirmed by visual observation of the wells indicates that, at higher doses (>5 μg ml$^{-1}$) for longer periods of time (>72 h), complete loss of cell viability occurs. In the clinical use of CsA as an immunosuppressive agent, a rapid initial phase of plasma clearance occurs following the attainment of peak levels of 1–2 μg ml$^{-1}$ (Kahan *et al.*, 1983). Levels during the prolonged second plateau phase are very much lower (~0.1 μg ml$^{-1}$). It appears unlikely therefore that the results which we report here in which continuous exposure to at least 1.0 μg ml$^{-1}$ was required for growth inhibition are of significance for the use of CsA as an anticancer agent. Nevertheless there is clearly a heterogeneity of sensitivity amongst lung cell lines and furthermore, OACsA in most of the lines studied is approximately 2-fold more potent than CsA.

Based on a report by Fidelius *et al.* (1984) that CsA could act as an inhibitor of orthinine decarboxylase and therefore of polyamine synthesis. Saydjari *et al.* (1986, 1987) investigated the *in vitro* effects of combining CsA with α-DFMO, a known inhibitor of polyamine synthesis. They studied the growth of hamster pancreatic and mouse colon carcinoma cells *in vitro* and at CsA doses of 1 and 5 $\mu g\ ml^{-1}$. In both series of experiments, a small inhibition of cell growth by CsA was reported which was both reversible by the addition of exogenous putrescine (0.05 mM) and synergistic with the effects of α-DFMO. In one of the studies the effects of CsA (1 $\mu g\ ml^{-1}$) upon polyamine levels were studied after 72 h exposure. Three and 2-fold reductions in levels of spermidine and spermine respectively were seen (Saydjari *et al.*, 1986). In contrast to these data, McLachlan *et al.* (1991) have shown that, in MOLT-4 T-lymphoblastic leukaemia cells, where a transient decrease in ODC activity was produced by exposure to CsA (2.5 or 5 $\mu g\ ml^{-1}$), there was no significant change in polyamine concentrations after 48 or 96 h of exposure. Moreover, growth inhibitory effects of CsA were unaffected by the addition of putrescine at concentrations between 0.1 and 10 mM. These data are therefore in agreement with the results which we present in this paper.

Following a description of its ability to act as a modifier of multidrug resistance (Slater *et al.*, 1986; Twentyman *et al.*, 1987) CsA was shown to bind to P-glycoprotein, the putative drug efflux pump molecule involved in the MDR phenotype (Foxwell *et al.*, 1989). It has also been demonstrated that MDR Chinese hamster cells accumulate less tritium-labelled CsA than the corresponding parent cells (Goldberg *et al.*, 1988). The clear reduction in CsA sensitivity seen in H69 LX4 (P-glycoprotein positive) compared with H69 P (P-glycoprotein negative) may therefore be accounted for by a similar differential CsA accumulation in this pair of cell lines. In our pairs of cell lines where the MDR phenotype occurs in the absence of P-glycoprotein, no such differential was seen.

The experiments which we have carried out do not clarify the mechanism whereby cyclosporins can inhibit cell growth. It is known that CsA binds to a specific cytosolic protein, cyclophilin (Hardschumacher *et al.*, 1984) and that this protein has peptidyl-prolyl cis-trans isomerase activity (Fischer *et al.*, 1989). It has been recently suggested that such interactions may interfere with the activity of protein kinase C and cell signal transduction (Tropschug & Hoffman, 1991). A number of groups have also reported effects of CsA on the physico-chemical properties of the plasma membrane. The drug binds to phospho-lipid vesicles, thereby disrupting membrane architecture (Haynes *et al.*, 1985) and interferes with the incorporation of fatty acids into the plasma membrane phospholipids of activated T-cells (Szamel *et al.*, 1986). It also depolarises cytoplasmic membrane potentials (Matyus *et al.*, 1986).

In conclusion, therefore, we have shown that CsA and OACsA will each inhibit growth of human lung cancer cells *in vitro*, but only at doses in excess of those which are clinically achievable. OACsA is, however, more potent than the parent compound and as further analogues enter clinical trial as possible resistance modifiers therefore, the possibility that antitumour effects of the cyclosporin alone may occur should be borne in mind.

**References**

BAILLIE-JOHNSON, H., TWENTYMAN, P.R., FOX, N.E., WALLS, G.A., WORKMAN, P., WATSON, J.V., JOHNSON, N., REEVE, J.G. & BLEEHEN, N.M. (1985). Establishment and characterisation of cell lines from patients with lung cancer (predominantly small cell carcinoma). *Br. J. Cancer*, **52**, 495–504.

BOREL, J.F., FEURER, C., GUBLER, H.U. & STAHELIN, H. (1976). Biological effects of cyclosporin A: a new antilymphocytic agent. *Agents Actions*, **6**, 468–475.

CHAMBERS, S.K., HAIT, W.N., KACINSKI, B.M., KEYES, S.R. & HANDSCHUMACHER, R.E. (1989). Enhancement of anthracycline growth inhibition in parent and multidrug-resistant Chinese hamster ovary cells by cyclosporin A and its analogues. *Cancer Res.*, **49**, 6275–6279.

COURTENAY, V.D. & MILLS, J. (1978). An *in vitro* colony assay for human tumours grown in immune suppressed mice and treated *in vivo* with cytotoxic agents. *Br. J. Cancer*, **37**, 261–268.

ELLIOTT, J.F., LIN, Y., MIZEL, S., BLEAKELY, R.E., HARNISH, D.G. & PRAETKAU, V. (1984). Induction of interleukin 2 messenger RNA inhibited by cyclosporin A. *Science*, **226**, 1439–1441.

FIDELUS, R.K., LAUGHER, A.H., TWOMEY, J.J., TAFFET, S.M. & HADDOX, M.K. (1984). The effect of cyclosporine on ornithine decarboxylase induction with mitogens, antigens, and lymphokines. *Transplant.*, **37**, 383–387.

FISHER, G., WITTMANN, L.B., LANG, K., KIEFHABER, T. & SCHMID, F.X. (1989). Cyclophilin and peptidyl-prolyl *cis-trans* isomerase are probably identical proteins. *Nature*, **337**, 476–478.

FOA, P., MAIOLO, A.T., BALDINI, L., MAISTO, A., SPANO, M., STARACE, G., QUARTO DI PALO, F. & POLLI, E.E. (1981). Antiproliferative activity of cyclosporin A on human T-lymphoblastic leukaemia cell line. *Lancet*, **i**, 838.

FOXWELL, B.M.J., MACKIE, A., LING, V. & RIFFEL, B. (1989). Identification of the multidrug resistance-related P-glycoprotein as a cyclosporine binding protein. *Molec. Pharmacol.*, **36**, 543–546.

GOLDBERG, H., LING, V., WONG, P.Y. & SKORECKI, K. (1988). Reduced cyclosporin accumulation in multidrug-resistant cells. *Biochem. Biophysic. Res. Comm.*, **152**, 552–558.

HANDSCHUMACHER, R.E., HARDING, M.W., RICE, J., DRUGGE, R.J. & SPEICHER, D.W. (1984). Cyclophilin: a specific cytosolic binding protein for cyclosporin A. *Science*, **226**, 544–547.

HAYNES, M., FULLER, L., HAYNES, D.H. & MILLER, J. (1985). Cyclosporin partitions into phospholipids vesicles and disrupts membrane architecture. *Immunol. Let.*, **11**, 343–349.

HESS, A.D., TUTSCHKA, P.J. & SANTOS, G.W. (1981). Effect of cyclosporin A on human lymphocyte responses *in vitro*. *J. Immunol.*, **126**, 961–968.

JABBAR, S.A.B., TWENTYMAN, P.R. & WATSON, J.V. (1989). The MTT assay underestimates the growth inhibitory effects of interferons. *Br. J. Cancer*, **60**, 523–528.

KREIS, W. & SORICELLI, A. (1979). Cyclosporins: immunosuppressive agents with antitumour activity. *Experientia*, **35**, 1506–1508.

KAHAN, B.D., REID, M. & NEWBURGER, J. (1983). Pharmacokinetics of cyclosporine in human renal transplantation. *Transplant*, **XV**, 446–453.

McLACHLAN, G., THOMSON, A.W. & WALLACE, H.M. (1991). Effects of cyclosporin A on growth and polyamine metabolism of MOLT-4 T-lymphoblastic leukaemia cells. *Br. J. Cancer*, **64**, 255–258.

MAYTUS, L., BALAZS, M., ASZALOS, A., MULHERN, S. & DAMJANOVIUCH, S. (1986). Cyclosporin A depolarizes cytoplasmic membrane potential and interacts with $Ca^{2+}$ ionophores. *Biochim. Biophys. Acta.*, **886**, 353–360.

MEADOR, J., SWEET, P., STUPECKY, M., WETZEL, M., MURRAY, S., GUPTA, S. & SLATER, L. (1987). Enhancement by cyclosporin A of daunorubicin efficacy in Ehrlich ascites carcinoma and murine hepatoma 129. *Cancer Res.*, **47**, 6216–6219.

MORELAND, A.A., ROBERTSON, D.B. & HEFFNER, L.T. (1985). Treatment of cutaneous T-cell lymphoma with cyclosporin A. *J. Amer. Acad. Dermat.*, **12**, 886–887.

MOSMANN, T. (1983). Rapid colorimetric assay for cellular growth and survival: application to proliferation and cytotoxicity assays. *J. Immunol. Meth.*, **65**, 55–63.

PRINCE, H.E. & JOHN, J.K. (1986). Cyclosporine inhibits the expression of receptors for interleukin 2 and transferrin on mitogen-activated human T lymphocytes. *Immun. Invest.*, **15**, 463–472.

PUTTICK, L., POLLACK, A. & FAIRBURN, E. (1983). Treatment of Sezary syndrome with cyclosporin A. *J. Roy. Soc. Med.*, **76**, 1063–1065.

REEVE, J.G., RABBITTS, P.H. & TWENTYMAN, P.R. (1989). Amplification and expression of mdr1 gene in a multidrug resistant variant of small cell lung cancer cell line NCI-H69. *Br. J. Cancer*, **60**, 339–342.

REEVE, J.G., RABBITTS, P.H. & TWENTYMAN, P.R. (1990). Non-P-glycoprotein-mediated multidrug resistance with reduced EGF receptor expression in a human large cell lung cancer cell line. *Br. J. Cancer*, **61**, 851–855.

SAYDJARI, R., TOWNSEND, C.M., BARRANCO, S.C., JAMES, E. & THOMPSON, J.C. (1986). Effects of cyclosporin and α-difluoromethylornithine on the growth of hamster pancreatic cancer *in vitro*. *J. Natl Cancer Inst.*, **77**, 1087–1092.

SAYDJARI, R., TOWNSEND, C.M., BARRANCO, S.C. & THOMPSON, J.C. (1987). Effects of cyclosporin and α-difluoromethylornithine on the growth of mouse colon cancer *in vitro*. *Life. Sci.*, **40**, 359-366.

SLATER, L.M., SWEET, P., STUPECKY, M. & GUPTA, S. (1986). Cyclosporin A reverses vincristine and daunorubicin resistance in acute lymphatic leukaemia *in vitro*. *J. Clin. Invest.*, **77**, 1405-1408.

SONNEVELD, P. & NOOTER, K. (1990). Reversal of drug-resistance by cyclosporin A in a patient with acute myelocytic leukaemia. *Br. J. Haematol.*, **75**, 208-211.

SZAMEL, M., BERGER, P. & RESCH, K. (1986). Inhibition of T lymphocyte activation by cyclosporin A: interference with the early activation of plasma membrane phospholipid metabolism. *J. Immunol.*, **136**, 264-269.

TOTTERMAN, T.H., DANERSUND, A., NILSON, K. & KILLANDER, A. (1982). Cyclosporin A is selectively cytotoxic to human leukaemic T-cell *in vitro*. *Blood*, **59**, 1103-1107.

TROPSCHUG, M. & HOFFMAN, R. (1991). FK506 and protein kinase C. *Nature*, **351**, 195.

TWENTYMAN, P.R. (1988). Resistance modification by non-immunosuppressive cyclosporins. *Br. J. Cancer*, **57**, 254-258.

TWENTYMAN, P.R., FOX, N.E. & WHITE, D.J.G. (1987). Cyclosporin A and its analogues as modifiers of adriamycin and vincristine resistance in a multi-drug resistant human lung cancer cell line. *Br. J. Cancer*, **56**, 55-57.

TWENTYMAN, P.R., FOX, N.E., WRIGHT, K.A. & BLEEHEN, N.M. (1986). Derivation and preliminary characterisation of adriamycin resistant lines of human lung cancer cells. *Br. J. Cancer*, **53**, 529-537.

WALLACE, H.M., GORDON, A.M., KEIR, H.M. & PEARSON, C.K. (1984). Activation of ADP-ribosyltransferase in polyamine depleted mammalian cells. *Biochem. J.*, **219**, 211-221.

WALLACE, H.M., NUTTALL, M.E., & ROBINSON, F.C. (1988). Acetylation of spermidine and methylglyoxal bis(guanylhydrazone) in baby hamster kidney cells (BHK 21 C13). *Biochem. J.*, **253**, 223-227.

WALLS, G.A. & TWENTYMAN, P.R. (1985). Cloning of human lung cancer cells. *Br. J. Cancer*, **52**, 505-513.

YANAGIHARA, R.H. & ADLER, W.H. (1983). Direct antiproliferative effects of cyclosporin A on murine lymphoreticular tumor cells in culture. *J. Biol. Resp. Mod.*, **2**, 121-132.